# FAMILY CAREGIVER SOCIAL ISOLATION AND HEALTH: FINDINGS IN THE NATIONAL SURVEY OF CAREGIVING

**DOI:** 10.1093/geroni/igz038.1645

**Published:** 2019-11-08

**Authors:** Janet S Pohl, Janice F Bell, Nancy Woods, Daniel J Tancredi

**Affiliations:** 1 Betty Irene Moore School of Nursing at University of California Davis, Sacramento, California, United States; 2 Betty Irene Moore School of Nursing, University of California Davis, Sacramento, California, United States; 3 University of Washington School of Nursing, Seattle, Washington, United States; 4 University Of California Davis, School Of Medicine, Sacramento, California, United States

## Abstract

Social relationships are important for family/informal caregiver health. Due to caregiving commitments caregivers are at risk for social isolation. Social isolation is associated with adverse health outcomes in the general population, but few studies have examined this association among caregivers. Employing the Convoy Model of social relations, we examined associations between caregiver self-reported health and social isolation—operationalized as a measure that included multiple domains and as specific domains modeled independently. Social isolation prevalence was 24.74% (n=2,175); mean health was 3.46 (SE=0.02) on a scale of 1 (poor) to 5 (excellent). In adjusted models, domain-inclusive social isolation was inversely associated with health (β=-.07; CI=-0.14,-0.02, p=0.01). In independent domain adjusted models, only participation in club activities was associated with health (β=-.22; CI=-0.35,-0.10, p<0.01). Social isolation predicted caregiver health, with club participation explaining much of the variance. Domain inclusive social isolation measures can identify targets for intervention studies to improve caregiver health.

